# The Association Between BMI and Depression Symptoms Among College Students: A Study Based on Random Intercept Cross-Lagged Model

**DOI:** 10.3390/bs16071058

**Published:** 2026-06-26

**Authors:** Jiahao Wang, Rong Fan, Jinbo Hou, Yin Fang

**Affiliations:** 1 School of Physical Education, China University of Geosciences (Wuhan), Wuhan 430074, China; wangjiahao@cug.edu.cn (J.W.); fy214@cug.edu.cn (Y.F.); 2 Psychological Center, China University of Geosciences (Wuhan), Wuhan 430074, China; houjinbo@cug.edu.cn

**Keywords:** body mass index, depressive symptoms, college student, random intercept cross-lag

## Abstract

**Objective:** To explore the longitudinal association between BMI and depressive symptoms among college students and to explore whether this association differs by gender. **Method:** A quantitative longitudinal design was adopted, and 1336 college students were tracked for a period of three years. The SCL-90 and BMI calculated from objectively measured height and weight were used to assess depressive symptoms and body mass status among college students. Using random intercept cross-lagged model analysis, we analyzed the temporal lagged associations between BMI and depressive symptoms and constructed stratified models for different genders to examine differences in these associations. **Result:** The results showed that there was a negative bidirectional predictive effect between BMI and depressive symptoms. Within-person deviations in depressive symptoms at T1 were significantly and negatively associated with deviations in BMI at T2 (*β* = −0.126, *p* = 0.003). The path from T2 to T3 was also significant (*β* = −0.079, *p* = 0.004). The BMI fluctuation at T1 could significantly negatively predict the depressive symptoms fluctuation at T2 (*β* = −0.133, *p* = 0.004). The path from T2 to T3 was also significant (*β* = −0.149, *p* = 0.003). At the interpersonal level, there was a significant positive correlation between RI_X and RI_Y (*β* = 0.142, *p* = 0.012). This means that, at the individual difference level, individuals with higher stable BMI traits also tend to have higher stable depressive symptoms traits. At the intra-individual level, the correlation between the BMI fluctuation and the depressive symptoms fluctuation at the same time point was not significant. Gender differences suggested that the longitudinal association between BMI and depressive symptoms may differ by gender. The BMI–depressive symptoms dynamics among male students appeared more complex. **Conclusions:** In the college student population, the association between BMI and depressive symptoms mainly reflects stable individual differences rather than continuous intra-individual dynamic effects. These findings highlight the value of separating between-person differences from within-person change when studying the physical and mental health of college students.

## 1. Introduction

Depressive disorder is a psychological health issue that has received sustained attention. According to data from the World Health Organization (World Health Organization, 2025), depressive symptoms affect 5% of adults worldwide and are associated with various diseases such as obesity and diabetes. Relevant reviews have shown that when obese young adults experience body dissatisfaction, their self-esteem decreases significantly, and the likelihood of experiencing more depressive symptoms increases (Moradi et al., 2021; Quek et al., 2017). Although the university stage is no longer the beginning of adolescence, it remains a period characterized by heightened sensitivity to self-evaluation, peer comparison, and intense pressure from appearance norms. Obesity is influenced by many factors, including lifestyle factors such as diet and physical activity (Cao et al., 2022; Quirk et al., 2013). Body mass index (BMI), as an objective physiological indicator, can directly reflect an individual’s health status and weight status. However, the relationship between BMI and depressive symptoms has not yet been fully clarified (Plackett, 2022). Therefore, further longitudinal research is needed to clarify the temporal association between BMI and depressive symptoms.

Current empirical findings regarding the relationship between BMI and depressive symptoms show substantial divergence. First, one view suggests a linear positive correlation, which suggests that higher body weight may increase exposure to body dissatisfaction, social appearance concerns, peer comparison, weight-related stigma, and internalized weight bias (Badillo et al., 2022; Dong et al., 2025; Ma et al., 2024). These experiences may contribute to negative self-evaluation and psychological distress, thereby increasing vulnerability to depressive symptoms (Haines & Neumark-Sztainer, 2006). From a psychosocial perspective, higher body weight may be associated not only with external weight stigma, but also with internalized weight bias, body dissatisfaction, social appearance anxiety, and negative self-evaluation. Recent evidence further suggests that weight self-stigma is related to emotional eating and diet satisfaction among individuals with obesity (Dilsiz & Arslan, 2023). Relevant studies indicate that this relationship may be bidirectional, meaning that depressive symptoms may increase an individual’s risk of obesity, while obesity may also inversely predict the occurrence of depressive symptoms (Mannan et al., 2016a). Second, some studies have shown that there is a nonlinear association between BMI and depressive symptoms. A high BMI does not necessarily indicate a greater risk of experiencing depressive symptoms, because diet, sedentary behavior, fitness needs, and other factors may lead to high BMI, and differences in muscle mass may even help reduce depressive symptoms (Mannan et al., 2016b; Magnusson et al., 2006). Beyond general associations between BMI and depressive symptoms, behavioral mechanisms may help explain why body weight is linked to psychological distress. Eating behavior regulation may be an important behavioral pathway connecting body weight and psychological distress. For example, emotional eating, uncontrolled eating, and cognitive restraint may reflect attempts to regulate negative emotions or manage body shape under appearance-related pressure, and food and cooking skills have been shown to be associated with eating behavior patterns among individuals with overweight or obesity (Arslan et al., 2023). Finally, some studies have also pointed out that the relationship between BMI and depressive symptoms remains unclear, and there is insufficient evidence to indicate a significant association between the two, possibly because BMI–depressive symptoms associations operate through multiple psychosocial and behavioral pathways (Yu et al., 2022). The above studies suggest that the association between BMI and depressive symptoms is not always consistent. Most previous studies have not clearly distinguished between-person associations from within-person changes. The question of whether students with higher BMI than their peers tend to report higher depressive symptoms is different from the question of whether the same student reports more depressive symptoms after his or her BMI increases relative to his or her own usual level. When these two levels are conflated, stable individual differences, such as body-related self-evaluation, weight stigma, eating behavior tendencies, or broader health-related characteristics, may be mistaken for within-person developmental processes. This may partly explain why previous studies have produced positive, nonlinear, or nonsignificant findings. Simple cross-sectional correlations often make it difficult to provide a convincing explanation (Hammerton et al., 2014; Tan et al., 2025).

Depressive symptoms among different groups in China have shown a continuous upward trend, and their prevention and treatment have become a crucial public health issue at present (Li & Yuan, 2025). Against this broader trend, college students are in a critical developmental stage of transition from family supervision to self-management, with academic and social adaptation pressures substantially overlapping. Therefore, the widespread occurrence and trend toward a younger age when assessing depressive symptoms deserve particular attention (Hua & Sun, 2021). In addition, individual differences have received widespread attention. Relevant studies have pointed out that college students’ depressive symptoms and their recurrence may predict the risk of obesity more strongly in women than in men (Zhu et al., 2017). Since physical health is closely related to mental health, previous studies have explored the relationship between extreme BMI and mental health (Treviño-Alvarez et al., 2023). Severe depressive symptoms are significantly associated with BMI-defined obesity, and atypical severe depressive symptoms are associated with a higher probability of weight gain. One study used structural equation modeling (SEM) to analyze the linear association between BMI and depressive symptoms, concluding that higher BMI may lead to more severe depressive symptoms (Dragan & Akhtar-Danesh, 2007). In addition, analyses using logistic regression and generalized linear models showed that persistent obesity in adulthood increases the risk of depressive symptoms, and that BMI change patterns play a role in the association between depressive symptoms and all-cause mortality (Xu et al., 2025). In studies examining whether there is a nonlinear association between BMI and depressive symptoms, a U-shaped association was observed between BMI and the risk of depressive symptoms. Meanwhile, BMI and depressive symptoms showed a U-shaped association in the younger subgroup, whereas this association was not observed in the middle-aged and older subgroups (Cui et al., 2024).

The different research approaches described above have explored multiple contributing factors. A more critical issue is that many controversies may not merely stem from differences in samples or measurements, but rather from the fact that studies have inadvertently conflated issues at two levels, namely, “whether people with higher body weight have higher depressive symptoms” as a between-person difference and “whether the same individual has higher depressive symptoms when gaining weight” as a within-person change over time. Traditional CLPM cannot distinguish between inter-individual differences and intra-individual changes. The random-intercept cross-lagged model introduces random intercept, separating stable between-person differences in variables from dynamic within-person changes, and on this basis examines temporal associations at the within-person level (Hamaker et al., 2015). In a highly homogeneous group such as college students, stable individual characteristics, such as genetic and early environmental factors, may obscure the dynamic interactions between variables. Without separating stable between-person differences, researchers may mistakenly regard differences between individuals as developmental patterns of individual change over time, thereby leading to overinterpretation of between-person associations as within-person developmental processes. Based on this, the present study adopts the random-intercept cross- lagged model (RI-CLPM). By introducing a random intercept, this model can accurately separate stable differences between individuals, thereby examining the bidirectional dynamic relationship between BMI and depressive symptoms among college students over three years at a purified “within-person level.”

No consistent conclusion has been reached regarding the association between BMI and depressive symptoms, which may stem from the complex mechanisms through which multiple factors may moderate the relationship between the two. The biopsychosocial perspective provides a useful conceptual background for understanding the association between BMI and depressive symptoms. This model was proposed by Engel in 1977 (Engel, 1977), moving beyond the limitations of the traditional biomedical paradigm and emphasizing that physical and mental health and disease are jointly shaped by the interaction and integration of biological, psychological, and social factors. This framework has been widely applied in health-related research and is particularly suitable for understanding physical and mental health issues influenced by multiple factors (Buckner et al., 2021; Rodgers et al., 2020). From this perspective, body weight and depressive symptoms may be shaped by biological, psychological, behavioral, and social processes. For example, both higher and slightly lower BMI levels may affect physical and mental health through different physiological pathways, such as inflammatory responses and metabolic dysregulation (Elsenburg et al., 2023; Xiang et al., 2022). The present study did not directly measure the full range of biopsychosocial mechanisms, such as diet, physical activity, or social support. Therefore, the current study focused specifically on the longitudinal between-person and within-person associations between BMI and depressive symptom scores among college students, with gender included as a covariate.

Based on the theoretical framework described above and previous empirical findings, the present study proposed the following a priori hypotheses. H1: BMI and depressive symptoms would show significant temporal stability across the three annual assessments, indicating that both physical status and depressive symptoms have a certain degree of continuity during the college period. H2: In the between-person level, students with a more stable BMI level would also tend to report more stable levels of depressive symptoms, reflecting a positive association between relatively enduring physical and psychological health characteristics. H3: At the within-person level, BMI and depressive symptoms would show reciprocal longitudinal associations after controlling for stable between-person differences and autoregressive effects. H4: As an exploratory hypothesis, the longitudinal association between BMI and depressive symptoms may differ between male and female students. This study can not only deepen the theoretical understanding of the relationship between the two, but also provide empirical evidence for precise psychological intervention and negative emotion management in universities.

## 2. Materials and Methods

### 2.1. Participants

A total of nine colleges, including the College of Arts and Media, the College of Economics and Management, and the Medical College, were selected from a university in Hubei Province. From each college, three classes were randomly selected through class-stratified sampling, and questionnaire surveys were conducted in three rounds. The first round of the survey (T1) was conducted in October 2022, and 1687 questionnaires were collected; the second round of the survey (T2) was conducted in October 2023 and collected 1466 questionnaires, with a dropout rate of 13.1%; the third round of the survey (T3) was conducted in October 2024 and collected 1579 questionnaires. After the data from the three rounds were organized, the questionnaires with missing values or completion errors were excluded. Finally, a total of 1336 valid cases were obtained. Among them, there were 760 male students (56.89%) and 576 female students (43.11%). There were 584 only-child students (43.71%) and 752 non-only-child students (56.29%). Gender was coded as 1 = male and 2 = female in all analyses.

Of the 1687 students who completed the first wave, 351 participants were excluded due to unmatched longitudinal records, missing key variables, or invalid/careless responses, resulting in 1336 participants in the final analytic sample. Baseline characteristics of the included and excluded participants were compared using χ^2^ tests for categorical variables (gender, place of origin, only-child status, parental education). No significant differences were observed between included and excluded participants (Table 1), suggesting that attrition did not introduce systematic bias in the analytic sample. The primary RI-CLPM analyses were conducted using the MLR estimator in Mplus, which implements full information maximum likelihood (FIML) under the missing-at-random assumption, thereby retaining participants with partially observed data and mitigating potential attrition bias.

### 2.2. Measurement Tools

A questionnaire survey was conducted using the Symptom Checklist-90 developed by Derogatis (Derogatis & Cleary, 1977). The SCL-90 is a 90-item self-report symptom inventory designed to assess a broad range of psychological symptoms across nine primary symptom dimensions. In the present study, the depression dimension was used to assess depressive symptom severity among college students rather than to provide a clinical diagnosis of depressive disorder. This dimension contains 13 items, namely items 5, 14, 15, 20, 22, 26, 29, 30, 31, 32, 54, 71, and 79, covering affective, motivational, cognitive, and somatic aspects of depressive symptoms. Higher scores indicate more severe depressive symptoms. This scale has been repeatedly used internationally and has demonstrated good reliability and validity, for example, in previous studies (Tomioka et al., 2008; Zhang et al., 2023). In the present study, the Cronbach’s α coefficients for T1, T2, and T3 were 0.849, 0.849, and 0.853, respectively.

BMI was calculated from objectively measured height and weight rather than from self-reported data. All measurements were conducted in a designated physical fitness testing area at the university. Before data collection, the assessors received standardized training regarding equipment use, participant positioning, measurement reading procedures, and data recording. Height was measured using a calibrated stadiometer, and body weight was measured using a calibrated electronic weight scale. The equipment was checked before each testing session to ensure measurement accuracy.

During the assessment, participants were asked to wear light clothing, remove shoes, hats, heavy coats, and personal items, and stand naturally on the measuring equipment. For height measurement, participants stood upright with heels together, arms relaxed at the sides, and head positioned in the horizontal plane. For weight measurement, participants stood still in the center of the scale until the reading stabilized. Height and weight values were recorded by trained assessors rather than by participants themselves. BMI was then calculated as weight in kilograms divided by height in meters squared and was used as a continuous variable in the subsequent analyses.

### 2.3. Ethical Approval

The study was performed in accordance with the Declaration of Helsinki. The study involving human participants was reviewed and approved by the Ethics Committee of China University of Geosciences (Wuhan). Written informed consent was obtained from all individual participants included in the study. Informed consent was obtained from school administrators, parents, and students. Participation was voluntary, and anonymity was guaranteed to reduce social desirability bias.

### 2.4. Statistical Analysis

Preliminary analyses were conducted in SPSS 27.0, including independent-samples *t*-tests for gender differences, descriptive statistics, and correlation analyses. Missing data were addressed using the MLR estimator in Mplus, which implements full information maximum likelihood under the missing-at-random assumption. Participants with partially missing longitudinal data were retained in the estimation, reducing the possibility that parameter estimates were biased by complete-case deletion. For descriptive purposes, participants with unmatched records, missing key variables, or careless responses were excluded, resulting in a complete-case sample of 1336 participants. Baseline characteristics of the included and excluded participants were compared using χ^2^ tests for categorical variables, and no significant differences were observed (*p* > 0.05), indicating that attrition did not introduce systematic bias. Sensitivity analyses using alternative RI-CLPM specifications confirmed that the substantive results were robust. Gender was included as a covariate in the model. Specifically, the random intercepts of all study variables were regressed on gender to adjust for stable between-person differences. Following the modeling procedure (Orth et al., 2021), a series of model constraints were applied to obtain the final model, with the specific constraint process presented in Table 2. Based on the final model, the association between BMI and depressive symptoms was examined. Because the RI-CLPM was estimated using observed composite scores of the SCL-90 depression dimension, formal longitudinal measurement invariance testing was not conducted. Therefore, the longitudinal paths involving depressive symptoms should be interpreted as prospective associations among observed depressive symptom scores rather than as evidence of changes in an invariant latent depressive symptom construct.

Because BMI was calculated from objectively measured height and weight, whereas depressive symptoms were assessed using the self-reported SCL-90, the two core variables were obtained from different measurement sources. This design reduced the likelihood that the observed association between BMI and depressive symptoms was mainly driven by common self-report bias (Podsakoff et al., 2003; Williams & McGonagle, 2016). Nevertheless, minor measurement error related to posture, clothing, or equipment variation cannot be completely ruled out, and this issue has been acknowledged as a limitation of the study.

## 3. Results

### 3.1. Descriptive Statistics of Variables

Consistent with H1, BMI and depressive symptom scores showed high stability across the three waves, as indicated by significant loadings on the respective random intercepts and strong autoregressive paths. The means, standard deviations, and correlation matrix of the main variables are shown in Table 3. BMI and depressive symptoms showed good temporal stability across the three waves of measurement, with all autocorrelations significant at *p* < 0.001. However, the cross-variable correlations between the two variables across different time points were generally weak and nonsignificant, with only T2 BMI showing a weak positive correlation with only T2 BMI showing a weak positive correlation with T1 depressive symptoms (r = 0.055). Gender was coded as 1 = male and 2 = female. Gender was significantly and negatively correlated with BMI and significantly and positively correlated with depressive symptom scores. Given this coding scheme, these results indicate that female students had lower BMI and higher depressive symptom scores than male students.

Correlation analyses may obscure the more complex dynamic relationship between BMI and depressive symptoms. First, cross-sectional correlations cannot distinguish stable individual differences from short-term fluctuations within individuals; second, they cannot test the longitudinal pathways through which variables predict each other. Therefore, to reveal the separated and temporally lagged dynamic process between the two at the within-person level, the random-intercept cross-lagged model was used for subsequent analysis, as this model can effectively separate between-person and within-person effects.

### 3.2. The Results of the Random Intercept Cross-Lag Panel Model

Model selection was based on transparent statistical and theoretical criteria, including absolute model fit, parsimony, the equal annual spacing of the three measurement waves, and the theoretical plausibility of time-invariant within-person processes (Table 2). The statistical significance of individual cross-lagged paths was not used as a model selection criterion. Model 2 showed the best overall fit among the constrained models. Although its fit indices, χ^2^(5) = 22.378, RMSEA = 0.051, CFI = 0.995, and SRMR = 0.056, showed a statistically significant deterioration compared with Model 2, Δχ^2^(2) = 11.753, *p* = 0.003, all overall fit indices remained within an acceptable range. Model 4 retained acceptable absolute fit indices and imposed additional stationarity constraints on the lagged paths and within-time residual covariances, making it the most parsimonious theoretically constrained specification. Therefore, Model 4 was retained as the primary model for substantive interpretation. Importantly, sensitivity analyses showed that the negative bidirectional cross-lagged associations were also observed in Model 2 (Table 4), indicating that the substantive findings were not unique to the final constrained model.

**Figure 1:**
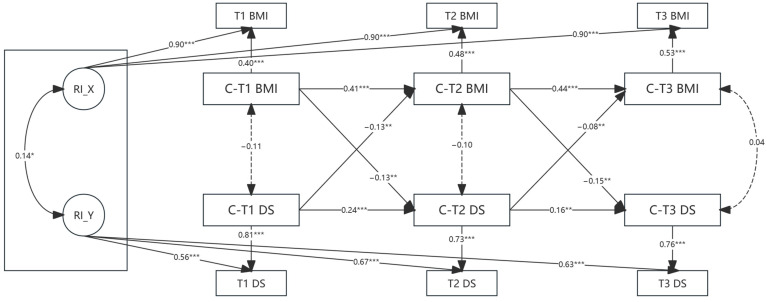
The full model of random intercept cross-lag between BMI and depressive symptoms (DS). * *p* < 0.05, ** *p* < 0.01, *** *p* < 0.001.

As shown in Figure 1, the RI-CLPM model can examine the longitudinal association between BMI and depressive symptoms at the within-person level. The final RI-CLPM had a statistically significant chi-square test, χ^2^(5) = 22.378, *p* < 0.001. However, given the sensitivity of the chi-square statistic to sample size, model fit was evaluated using multiple fit indices. The fit indices indicated acceptable overall fit: RMSEA = 0.051, CFI = 0.995, TLI = 0.979, and SRMR = 0.056. The standardized parameter estimates were as follows, with all reported path coefficients being standardized estimates: (1) The results at the between-person level showed that the factor loadings of the BMI random intercept, RI_X, across the three waves were 0.893, 0.853, and 0.806, respectively, *p* < 0.001; the factor loadings of the depressive symptoms random intercept, RI_Y, were 0.557, 0.673, and 0.631, respectively, *p* < 0.001. These high and statistically significant loadings indicate that the model successfully extracted relatively stable trait components of BMI and depressive symptoms among individuals. There was a significant positive correlation between RI_X and RI_Y (β = 0.142, *p* = 0.012), supporting H2. This means that, at the level of individual differences, individuals with higher stable BMI traits also tended to have higher stable depressive symptoms traits. (2) The autoregressive paths indicated that BMI and depressive symptoms showed a certain degree of stability across the three time periods, *p* < 0.001, suggesting that short-term fluctuations within individuals were relatively stable. (3) In terms of cross-lagged effects, after controlling for stable between-person differences and autoregressive effects, a significant bidirectional negative cross-lagged associations between BMI and depressive symptoms was observed at the within-person level, supporting H3. Within-person deviations in depressive symptoms at T1 were significantly and negatively associated with deviations in BMI at T2, β = −0.126, *p* = 0.003. The path from T2 to T3 was also significant, β = −0.079, *p* = 0.004. This suggests that when an individual reported higher-than-usual depressive symptoms at a given time point, their BMI tended to be lower than usual at the subsequent time point. BMI fluctuations at T1 significantly and negatively predicted depressive symptoms fluctuations at T2, β = −0.133, *p* = 0.004. The path from T2 to T3 was also significant, β = −0.149, *p* = 0.003. This indicates that when an individual’s BMI is higher than usual at a certain time point, their depressive symptoms at the next time point tended to be lower than usual. (4) After controlling for stable traits and the effects of the previous time point, the correlations between within-person BMI fluctuations and fluctuations in depressive symptoms at the same time point were not significant. The specific indicators of autoregressive paths, cross-lagged paths, within-time residual correlations, and random intercept correlations are presented in Supplementary Material S1.

### 3.3. Exploratory Gender-Stratified Analysis

**Figure 2:**
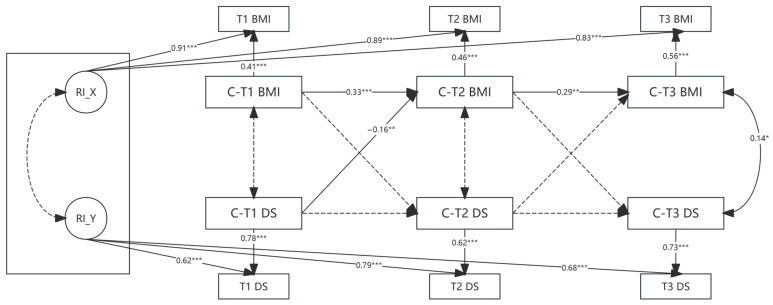
A random intercept cross-lag model of female BMI and depressive symptoms (DS). * *p* < 0.05, ** *p* < 0.01, *** *p* < 0.001.

**Figure 3:**
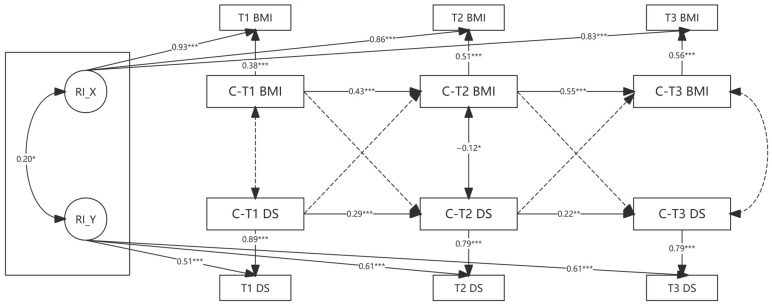
A random intercept cross-lag model of male BMI and depressive symptoms (DS). * *p* < 0.05, ** *p* < 0.01, *** *p* < 0.001.

Gender was coded as 1 = male and 2 = female. In the overall model, gender significantly and negatively predicted BMI across the three waves, with β values ranging from −0.193 to −0.257, *p* < 0.001, and significantly and positively predicted depressive symptoms, with β values ranging from 0.142 to 0.164, *p* < 0.001. Given the coding scheme, these results indicate that female students had lower BMI and higher depressive symptom scores than male students. This analytic strategy is consistent with previous methodological recommendations, which suggest that when full invariance cannot be established, separate group models can provide valuable descriptive and inferential insights into group-specific developmental processes (Little, 2024; Mulder & Hamaker, 2021). Exploratory gender-stratified RI-CLPMs were further estimated. The female model showed acceptable model fit, χ^2^(4) = 7.029, *p* = 0.134, RMSEA = 0.036, 90% CI [0.002, 0.068], CFI = 0.990, TLI = 0.986, and SRMR = 0.062 (Figure 2). In contrast, the male model showed suboptimal fit, χ^2^(4) = 41.382, *p* < 0.001, RMSEA = 0.110, 90% CI [0.074, 0.129], CFI = 0.980, TLI = 0.939, and SRMR = 0.116 (Figure 3). Although CFI remained high, the RMSEA and SRMR values suggested that the BMI–depressive symptoms dynamics among male students appeared more complex. No strong conclusions were drawn regarding male-specific longitudinal dynamics between BMI and depressive symptom scores. H4 received exploratory support.

## 4. Discussion

Understanding the temporal dynamics between BMI and depressive symptoms is crucial for exploring the developmental pathways of college students’ internalizing psychological problems. However, existing studies have often conflated between-person associations with within-person associations and have insufficiently distinguished these two levels of influence. This study examined the temporal ordering of the association between depressive symptoms and BMI among Chinese college students through the random-intercept cross-lagged model, thereby extending existing research. The results showed that lower-than-usual depressive symptoms were associated with higher-than-usual BMI at the within-person level in the subsequent wave, and that BMI was negatively predicted depressive symptoms in the subsequent time period. The findings support the presence of between-person differences in BMI and depressive symptoms, regardless of their underlying motivation.

### 4.1. The Association Between BMI and Depressive at the Inner Level of a Person

First, the autoregressive paths of BMI and depressive symptoms showed that when an individual’s BMI at T1 was higher than his or her long-term average level, this elevated state still showed a moderate degree of continuity in the following year, with BMI autoregressive path from T1 to T2 being 0.41*** and BMI from T2 to T3 being 0.44***. Meanwhile, short-term deviations in depressive symptoms also showed significant but relatively weaker temporal continuity, with depressive symptoms’ autoregressive path from T1 to T2 being 0.24*** and depressive symptoms from T2 to T3 being 0.16***. In longitudinal research on mental health, fluctuations in depressive symptoms are more malleable than BMI and are strongly and complexly influenced by environmental, social, and other factors, which is consistent with existing research findings (Eik-Nes et al., 2022). Second, regarding the cross-lagged associations, the results indicated that within-person deviations in BMI were prospectively associated with subsequent deviations in depressive symptom scores. When college students’ BMI was higher than their own usual level at one wave, they tended to report lower depressive symptom scores at the following wave. These associations reflect within-person dynamics rather than between-person differences or causal effects. Specifically, higher- than-usual BMI at T1 was associated with lower depressive symptoms at T2, with β = −0.13 and *p* < 0.01, and a similar pattern was also observed from T2 to T3, with β = −0.08 and *p* < 0.01. Although the cross-lagged associations were statistically significant, their standardized coefficients were modest in magnitude. Therefore, these findings should not be interpreted as evidence of large individual-level clinical effects. However, the modest size of the effects does not negate their theoretical and public health relevance. In longitudinal RI-CLPM analyses, cross-lagged paths are estimated after separating stable between-person differences and controlling for prior levels of the same variable; therefore, even modest within-person associations may provide useful information about temporal coupling between physical and psychological health indicators. In this sense, the present findings are more relevant to understanding population-level developmental patterns and early risk monitoring among college students than to making individual-level clinical judgments.

These findings indicate that, after separating stable between-person differences, higher-than-usual BMI was followed by lower observed depressive symptom scores at the subsequent wave, and higher-than-usual depressive symptom scores were followed by lower BMI at the subsequent wave. This indicates a negative prospective within-person association between BMI and depressive symptoms. Several hypotheses may help explain this finding and should be examined in future research. First, a higher-than-usual BMI may, in some students, reflect improved nutritional status, more adequate energy supply, or more regular daily routines rather than unhealthy weight gain. These changes may be associated with better physical functioning and emotional regulation, which could partly explain the subsequent reduction in depressive symptom scores (Gao et al., 2024). Second, BMI increases may also occur alongside changes in physical activity or resistance training. For example, increased muscle mass or participation in mixed aerobic and resistance exercise may be accompanied by psychological benefits, such as improved self-efficacy, reduced anxiety, and better mood regulation (Magni et al., 2024). Third, the reverse pathway from higher-than-usual depressive symptoms to lower subsequent BMI may be hypothesized to involve reduced appetite, irregular eating, decreased motivation for exercise, disrupted sleep, or stress-related metabolic changes. The main contribution of this study is to show that the BMI–depressive symptoms association differs across levels of analysis: stable between-person differences showed a positive association, whereas within-person deviations showed negative prospective associations. Future studies should include direct measures of diet, physical activity, sleep, body composition, and lifestyle regularity to test these hypothesized mechanisms more formally.

However, these associations appeared only in the within-person pathways, whereas the concurrent correlation between BMI and depressive symptoms was not significant. This result also suggests that the dynamic interaction between BMI and depressive symptoms unfolds over time rather than occurring simultaneously. This means that interventions and approaches targeting physical and mental health may produce delayed cross-domain effects rather than immediate mutual influences, thereby highlighting the importance of sustained participation in physical activity and lifestyle interventions, because their psychological benefits may gradually emerge rather than producing immediate feedback.

### 4.2. The Stability of BMI and Depressive Among Individuals

This study adopted a random-intercept cross-lagged panel framework to model stable between person differences in BMI and depressive symptoms. The random intercept level can capture stable higher or lower levels of BMI and depressive symptoms over three years, distinguishing stable between person differences from within-person fluctuations. The results showed that the observed BMI and depressive symptoms had strong and significant loadings on the random intercepts at different time points over the three years, indicating that both had high between-person stability. The BMI random intercept was positively correlated with the depressive symptoms random intercept, suggesting that college students with stably higher BMI over the three years also tended to have higher depressive symptoms. This correlation reflects stable between-person differences rather than common fluctuations or temporal processes. The positive association at the between-person level may be understood within this psychosocial framework. Students with relatively stable higher BMI may also be more likely to experience stable body-related vulnerability, such as body dissatisfaction, appearance-related concerns, weight stigma, internalized weight bias, or eating behavior, which may be associated with persistently higher depressive symptoms. Future research should directly measure these factors to test whether these psychosocial and behavioral pathways can explain the level-specific associations observed in this RI-CLPM study.

The apparently opposite directions of the between-person and within-person associations are theoretically meaningful rather than contradictory. The positive association at the random-intercept level indicates that students who maintained relatively higher BMI than their peers over the three years also tended to maintain relatively higher depressive symptoms. The association between increased BMI and the development of depressive symptoms appears to emerge and remain stable over the long term, which is consistent with previous research findings (Gibson-Smith et al., 2016). This pattern may reflect stable biopsychosocial vulnerability, such as long-term differences in body-related self-evaluation, health status, lifestyle regularity, or exposure to weight-related psychosocial stress. However, the negative within-person cross-lagged paths describe a different process: whether temporary deviations from an individual’s own usual level predict later deviations in the other variable. In this sense, a higher-than-usual BMI at one wave does not have the same meaning as having stably higher BMI across years. It may instead represent a short-term change relative to one’s own baseline, which was followed by lower depressive symptoms at the next wave. Similarly, higher-than-usual depressive symptoms were followed by lower BMI at the next wave. Therefore, the findings suggest that stable between-person risk patterns and within-person dynamic fluctuations may operate through different mechanisms. This distinction is central to the RI-CLPM framework and helps explain why previous studies that did not separate between-person and within-person effects may have produced inconsistent conclusions regarding the BMI and depressive symptoms.

The gender-stratified analyses should be interpreted with caution. Although the female model showed good fit, the male model showed suboptimal fit, particularly in terms of RMSEA and SRMR. This pattern may suggest that the assumed RI-CLPM structure captured the BMI–depressive symptoms dynamics less adequately among male students than among female students. One possible explanation is that the association between BMI and depressive symptoms among male students may be influenced by additional unmeasured factors, such as physical activity patterns, social context, or stress exposure. However, because these factors were not included in the present model and the male-specific model fit was suboptimal, we do not interpret this finding as definitive evidence of greater heterogeneity among male students. Future studies should examine gender differences using larger samples, formal multi-group models, and additional contextual or behavioral covariates.

This study offers methodological innovations. First, data from three time periods allow examination of temporal ordering and potential bidirectional associations, which is meaningful for studying the relationship between stable BMI and depressive symptoms. Multiple time points allow examination of the dynamic relationships between variables, whereas cross-sectional studies usually require assumptions about temporal order. Second, RI-CLPM can control for unidentified and time-invariant individual differences. This modeling approach allows the observed associations to reflect within-person changes, rather than changes caused by factors such as personality or baseline health. Finally, the relatively large sample size and three-wave longitudinal design supported the internal robustness of the model estimation, and subgroup analyses were conducted to explore gender differences in the association between BMI and depressive symptoms.

Several limitations should also be considered. First, all students came from one university in Hubei Province. The observed BMI–depressive symptoms dynamics may therefore be influenced by institution-specific factors, such as campus environment and academic pressure. Future research should involve universities from different regions and socioeconomic backgrounds to examine whether the between-person and within-person associations are generalizable across contexts. Second, this study only examined gender differences in BMI and depressive symptoms; the gender-stratified analyses were exploratory, and BMI cannot distinguish between body fat and lean body mass. In addition, other potential factors should also be taken into account, such as social factors, academic stress, diet, and sleep. Future studies may consider incorporating these factors to establish a more complete theoretical model. Third, although BMI was not self-reported and was calculated from objectively measured height and weight, small measurement errors may still have occurred due to clothing, posture, or equipment-related factors. Finally, although the SCL-90 depression dimension showed acceptable reliability across waves, the present study did not conduct formal longitudinal measurement invariance testing. Therefore, it cannot be fully determined whether changes in observed depressive symptom scores across waves reflect only true changes in the underlying depressive symptom construct or whether they are partly influenced by changes in item interpretation or response patterns over time. Future studies should use item-level longitudinal measurement models to test configural, metric, and scalar invariance before examining latent within-person changes.

## 5. Conclusions

Although research on the association between BMI and depressive symptoms among college students is the result of multiple interacting factors, the inconsistency of research conclusions raises another issue. That is, whether between-person differences and within-person changes are the key source of this inconsistency. By using a random-intercept cross-lagged panel model, this study explored the within-person and between-person associations between BMI and depressive symptoms among Chinese college students. The results showed that, at the random intercept level, the correlation between the BMI and depressive symptoms random intercepts indicated that these two constructs were stable at the between-person level, whereas the concurrent correlation between BMI and depressive symptoms was not significant. This study suggests that the association between BMI and depressive symptoms may unfold over time rather than occur only concurrently. Meanwhile, the bidirectional cross-lagged effects between BMI and depressive symptoms indicated that higher-than-usual BMI was associated with a reduction in depressive symptoms or mood-related symptoms in the subsequent time period, and vice versa. These findings advance the understanding of the pathways of college students’ internalized problems and have practical significance for prioritizing screening for depressive symptoms and intervention to alleviate psychological symptoms and related behaviors.

## Figures and Tables

**Table 1:** Comparison of baseline characteristics of samples.

Information Characteristic	351 Samples		1336 Samples	
	χ^2^	*p*	χ^2^	*p*
only child	8.158	0.086	0.178	0.673
Place of origin	0.241	0.623	2.435	0.119
Father’s education	0.381	0.537	4.957	0.292
Mother’s education	2.051	0.726	2.108	0.716

Note: only child: 1 = yes, 2 = no; place of origin: 1 = urban, 2 = rural; parent’s education: 1 = primary school or below; 2 = junior high school; 3 = high school or technical school; 4 = bachelor’s or associate’s degree; 5 = graduate or above. *p* > 0.05.

**Table 2:** Model Comparison test of RI-CLPM on BMI and depressive symptoms.

Model	χ^2^ (df)	ΔCFL	ΔRMSEA	RMSEA [95%CI]	CFI	TLI	SRMR
MODEL1	0.158(1)			0.000 [0.000, 0.054]	1.00	1.00	0.002
MODEL2	10.625(3)	−0.002	0.044	0.044 [0.017, 0.073]	0.998	0.984	0.019
MODEL3	16.530(3)	−0.002	0.014	0.058 [0.033, 0.087]	0.996	0.972	0.054
MODEL4	22.378(5)	−0.001	0.007	0.051 [0.031, 0.073]	0.995	0.979	0.056

Note: MODEL1 = Unconstrained baseline Model; MODEL2 = A time-invariant model with all cross-lag paths fixed; MODEL3 = A time-invariant model with all time-varying parameters fixed; MODEL4 = A model that includes all cross-lag paths, all stability paths, and all T2-T3-related changes, with time remaining constant. ΔCFI and ΔRMSEA represent changes in CFI and RMSEA between nested models, respectively.

**Table 3:** Mean, standard deviation and correlation analysis of the main variables.

Dimension	M	SD	T1BMI	T2BMI	T3BMI	T1 DS	T2 DS	T3 DS	Gender
T1BMI	20.52	2.22	1						
T2BMI	20.77	2.26	0.876 **	1					
T3BMI	21.12	2.51	0.819 **	0.861 **	1				
T1 DS	1.51	0.49	0.004	−0.032	−0.023	1			
T2 DS	1.45	0.41	0.055 *	0.026	0.007	0.544 **	1		
T3 DS	1.38	0.43	−0.002	−0.051	−0.019	0.415 **	0.553 **	1	
Gender	1.43	0.50	−0.200 **	−0.199 **	−0.252 **	0.143 **	0.142 **	0.164 **	1

Note: M = mean; SD = standard deviation. * *p* < 0.05; ** *p* < 0.01. All tests were two-tailed. DS = depressive symptoms.

**Table 4:** Sensitivity analyses of key parameter estimates across alternative RI-CLPM specifications.

Model	RI_X–RI_Y	T1 BMI-T2 DS	T2 BMI-T3 DS	T1 DS-T2 BMI	T2 DS-T3 BMI
Model 1	0.01	0.15	−0.06	−0.09	0.17
Model 2	0.10	−0.10 **	−0.11 **	−0.19 **	−0.08 *
Model 3	0.05	0.18	−0.17 **	−0.08	0.01
Model 4	0.14	−0.13 **	−0.15 **	−0.13 **	−0.08 *

Note: Values are standardized coefficients. * *p* < 0.05, ** *p* < 0.01. RI_X–RI_Y represents the correlation between the random intercepts of BMI and depressive symptoms. DS = depressive symptoms.

## Data Availability

The data presented in this study are available on request from the corresponding author. The data are not publicly available due to ethical restrictions.

## Supplementary Materials

The following supporting information can be downloaded at: https://www.mdpi.com/article/10.3390/bs16071058/s1.
